# A novel fluorescence-activated cell sorting (FACS)-based screening identified *ATG14*, the gene required for pexophagy in the methylotrophic yeast

**DOI:** 10.1093/femsyr/foae022

**Published:** 2024-07-18

**Authors:** Kosuke Shiraishi, Yumi Arima, Motoharu Nakamura, Takumi Nakatsuji, Masahide Oku, Yasuyoshi Sakai

**Affiliations:** Division of Applied Life Sciences, Graduate School of Agriculture, Kyoto University, Kitashirakawa-Oiwake, Sakyo-ku, Kyoto 606-8502, Japan; Division of Applied Life Sciences, Graduate School of Agriculture, Kyoto University, Kitashirakawa-Oiwake, Sakyo-ku, Kyoto 606-8502, Japan; Division of Applied Life Sciences, Graduate School of Agriculture, Kyoto University, Kitashirakawa-Oiwake, Sakyo-ku, Kyoto 606-8502, Japan; Division of Applied Life Sciences, Graduate School of Agriculture, Kyoto University, Kitashirakawa-Oiwake, Sakyo-ku, Kyoto 606-8502, Japan; Department of Bioscience and Biotechnology, Faculty of Bioenvironmental Science, Kyoto University of Advanced Science, Otani 1-1, Sogabecho Nanjo, Kameoka 621-0023, Japan; Division of Applied Life Sciences, Graduate School of Agriculture, Kyoto University, Kitashirakawa-Oiwake, Sakyo-ku, Kyoto 606-8502, Japan

**Keywords:** FACS, pexophagy, autophagy, Atg14, methylotrophic yeast

## Abstract

Pexophagy is a type of autophagy that selectively degrades peroxisomes and can be classified as either macropexophagy or micropexophagy. During macropexophagy, individual peroxisomes are sequestered by pexophagosomes and transported to the vacuole for degradation, while in micropexophagy, peroxisomes are directly engulfed by the septated vacuole. To date, some autophagy-related genes (*ATGs*) required for pexophagy have been identified through plate-based assays performed primarily under micropexophagy-induced conditions. Here, we developed a novel high-throughput screening system using fluorescence-activated cell sorting (FACS) to identify genes required for macropexophagy. Using this system, we discovered *KpATG14*, a gene that could not be identified previously in the methylotrophic yeast *Komagataella phaffii* due to technical limitations. Microscopic and immunoblot analyses found that KpAtg14 was required for both macropexophagy and micropexophagy. We also revealed that KpAtg14 was necessary for recruitment of the downstream factor KpAtg5 at the preautophagosomal structure (PAS), and consequently, for bulk autophagy. We anticipate our assay to be used to identify novel genes that are exclusively required for macropexophagy, leading to better understanding of the physiological significance of the existing two types of autophagic degradation pathways for peroxisomes.

## Teaser

This paper describes a newly established FACS-based high-throughput screening system to identify genes required for macropexophagy and a novel gene discovered through that method for the first time.

## Introduction

Autophagy is an intracellular degradation system that is highly conserved among eukaryotes. In yeast species, autophagy can be classified as macroautophagy or microautophagy. In macroautophagy, autophagosomes separated by bilayers sequester the cytoplasmic cargo and fuse with the vacuole for degradation (Suzuki et al. 2001, Suzuki and Ohsumi 2007). Microautophagy, in contrast, involves the direct engulfment of the target cargo by the vacuole. During the process, the vacuolar membrane randomly invaginates and differentiates into the autophagy tube (Wang et al. 2022). Autophagy pathways can be further divided into nonselective or selective types. Nonselective autophagy involves the random delivery of a portion of the cytoplasm to the vacuole, whereas selective autophagy recognizes and degrades specific cargoes, e.g. organelles, aggregates, and pathogens (Lamark and Johansen 2021). These conserved processes are achieved through the coordination of different autophagy-related (ATG) proteins and more than 40 ATG proteins have been discovered to date (Ohsumi 2014).

Pexophagy is a selective autophagy pathway for the specific degradation of peroxisomes. Based on the vacuolar membrane dynamics, pexophagy is categorized into macropexophagy and micropexophagy (Oku and Sakai 2018). In the methylotrophic yeast *Komagataella phaffii* (synoym *Pichia pastoris*), macropexophagy is induced upon the carbon source shift from methanol to ethanol. Macropexophagy belongs to the macroautophagic process in which individual peroxisomes are sequestered by newly synthesized membrane structures called pexophagosomes (Tuttle and Dunn 1995). Micropexophagy, on the other hand, is induced after the medium transition from methanol to glucose. During the process, peroxisomes are directly engulfed by vacuolar sequestering membranes (VSMs) extending from the septated vacuole and a double-membrane structure called micropexophagy-specific membrane apparatus (MIPA) (Mukaiyama et al. 2004). The formation of both MIPA and pexophagosomes is mediated by ATG proteins (Oku and Sakai 2016). To date, several ATG proteins required for pexophagy have been identified by plate-based assays performed primarily under micropexophagy-inducing conditions (Tuttle and Dunn 1995, Sakai et al. 1998b, Mukaiyama et al. 2002, Dunn et al. 2005) as screening under macropexophagy-induced conditions has often yielded false positive results.

In recent years, fluorescence-activated cell sorting (FACS) has become a powerful method for screening specific gene mutants from large libraries. It is a specialized instrument that can query multiple fluorescence parameters of individual particles such as cells and microbeads at a rate of ~10^7^ per hour (Shapiro 2005). Taking full advantage of its high-throughput nature, the application of FACS screening has been expanded to various research fields including autophagy (Morita et al. 2018, Shoemaker et al. 2019).

In this study, we established a novel high-throughput FACS-based screening method to identify the genes required for macropexophagy. Through the screening, we discovered the *KpATG14* gene, which could not be identified for a long time due to technical limitations of existing methods. KpAtg14 showed relatively low similarity to ScAtg14 in the budding yeast *Saccharomyces cerevisiae* and SpAtg14 in the fission yeast *Schizosaccharomyces pombe*, whereas critical domains such as the cysteine repeats and coiled-coil domains at the N-terminus were conserved. Through microscopic and immunoblot analyses we revealed that KpAtg14 was required for both macro- and micropexophagy. In addition, we also found that KpAtg14 was necessary for recruiting downstream ATG proteins, and thus for bulk autophagy degradation, in a manner similar to that of ScAtg14.

## Materials and methods

### Yeast strain, culture conditions, and reagents

The yeast strains used in this work are listed in Table 1. Cells were grown at 28ºC on the appropriate media described below. Yeast extract, peptone, and dextrose (YPD) medium consisted of 1% yeast extract, 2% bactopeptone, and 2% glucose; synthetic dextrose (SD) medium contained 0.67% yeast nitrogen base without amino acids and 2% glucose; synthetic methanol (SM) medium had 0.67% yeast nitrogen base without amino acids and 0.7% methanol; synthetic ethanol (SE) medium consisted of 0.67% yeast nitrogen base without amino acids and 0.5% ethanol; and synthetic dextrose without nitrogen source (SD-N) medium contained 0.67% yeast nitrogen base without amino acids and ammonium sulfate and 2% glucose. Yeast extract, yeast nitrogen base without amino acids and dextrose (YND), yeast extract, yeast nitrogen base without amino acids and methanol (YNM) and yeast extract, yeast nitrogen base without amino acids and ethanol (YNE) media were made with the addition of 0.5% yeast extract to SD, SM, and SE media, respectively. These synthetic media were supplemented with the appropriate amino acids (100 µg/ml arginine and/or 100 µg/ml histidine) and/or antibiotics (100 µg/ml zeocin), as necessary. The pH of SD, SM, SE, YND, YNM, and YNE media was adjusted to 6.0 with NaOH. Growth was monitored by measuring the optical density at 610 nm (OD_610_). The nucleotide sequence of *KpATG14* was deposited in the DDBJ/EMBL/GenBank under accession number LC815816.

**Table 1.** Yeast strains used in this study.

Designation	Description	Genotype	References
PPY12	Wild type	*arg4 his4*	Sakai et al. (1998a)
TN0001	*Kpatg14∆*	*Kpatg14∆*::Zeo^r^ *arg4 his4*	This study
IS17020	*Kpatg30∆*	*Kpatg30∆*::Bsd^r^ *arg4 his4*	Ohsawa et al. (2021)
MN0001	KpPex11-pHluorin-mCherry PPY12	PPY12 *his4*::KpPex11-pHluorin-mCherry	This study
MN0002	KpPex11-pHluorin-mCherry *Kpatg14∆*	TN0001 *his4*::KpPex11-pHluorin-mCherry	This study
MN0003	KpPex11-pHluorin-mCherry *Kpatg30∆*	IS17020 *his4*::KpPex11-pHluorin-mCherry	This study
YAP0004	YFP-KpAtg8 CFP-SKL PPY12	PPY12 *arg4*::pSAP115 *his4*::pYA006	Ano et al. (2005)
ARM0001	YFP-KpAtg8 CFP-SKL *Kpatg14∆*	TN0001 *arg4*::YFP-KpAtg8 *his4*::CFP-SKL	This study
ARM0002	YFP-KpAtg8 CFP-SKL *Kpatg30∆*	IS17020 *arg4*::YFP-KpAtg8 *his4*::CFP-SKL	This study
MN0004	KpPex11-pHluorin PPY12	PPY12 *his4*::KpPex11-pHluorin	This study
ARM0003	KpPex11-pHluorin *Kpatg14∆*	TN0001 *his4*::KpPex11-pHluorin	This study
MN0005	KpPex11-pHluorin *Kpatg30∆*	IS17020 *his4*::KpPex11-pHluorin	This study
ARM0004	KpAtg14-Cerulean YFP-KpAtg8 PPY12	PPY12 *arg4*::KpAtg14-Cerulean *his4*::YFP-KpAtg8	This study
KS0001	KpIdh1-YFP PPY12	PPY12 *arg4*::KpIdh1-YFP	This study
KS0002	KpIdh1-YFP *Kpatg14∆*	TN0001 *arg4*::KpIdh1-YFP	This study
TN0002	KpPgk1-CFP PPY12	PPY12 *his4*::KpPgk1-CFP	This study
TN0003	KpPgk1-CFP *Kpatg14∆*	TN0001 *his4*::KpPgk1-CFP	This study
ARM0005	KpAtg5-Cerulean YFP-KpAtg8 PPY12	PPY12 *arg4*::KpAtg5-Cerulean *his4*::YFP-KpAtg8	This study
ARM0006	KpAtg5-Ceruelan YFP-KpAtg8 *Kpatg14∆*	IS17020 *arg4*::KpAtg5-Cerulean *his4*::YFP-KpAtg8	This study
ARM0007	KpAtg14-FLAG KpPgk1-CFP *Kpatg14∆*	TN0003 *arg4*::KpAtg14-FLAG	This study
ARM0008	KpAtg14∆C84-FLAG KpPgk1-CFP *Kpatg14∆*	TN0003 *arg4*::KpAtg14∆C84-FLAG	This study
ARM0009	KpAtg14-FLAG KpPex11-pHluorin *Kpatg14∆*	YA0003 *arg4*::KpAtg14-FLAG	This study
ARM0010	KpAtg14∆C84-FLAG KpPex11-pHluorin *Kpatg14∆*	YA0003 *arg4*::KpAtg14∆C84-FLAG	This study
ARM0011	KpAtg14-FLAG CFP-SKL *Kpatg14∆*	TN0003 *arg4*::KpAtg14-FLAG *his4*::CFP-SKL	This study
ARM0012	KpAtg14∆C84-FLAG CFP-SKL *Kpatg14∆*	TN0003 *arg4*::KpAtg14∆C84-FLAG *his4*::CFP-SKL	This study
ARM0013	KpAtg14-Cerulean YFP-KpAtg8 *Kpatg14∆*	TN0003 *arg4*::KpAtg14-Cerulean *his4*::YFP-KpAtg8	This study
ARM0014	KpAtg14∆C84-Cerulean YFP-KpAtg8 *Kpatg14∆*	TN0003 *arg4*::KpAtg14∆C84-Cerulean *his4*::YFP-KpAtg8	This study

### Plasmid construction and gene disruption

Primers used in this study are listed in Table 2, and plasmids used in this study are listed in Table 3. The plasmid vectors pREMI-Z (Mukaiyama et al. 2002), pIB1 (Sears et al. 1998), pNT204 (Tamura et al. 2010), SK + Zeo^r^ (Yano et al. 2010), PSAP115 (Mukaiyama et al. 2004), pYA006 (Ano et al. 2005), pNT812 (Tamura et al. 2010), pMO500 (Pang et al. 2019), pBW1679 (gifted by Dr Beverly Wendland) (Prosser et al. 2010), pmCherry (purchased from Clontech, Mountain View, CA, USA) (Maeda et al. 2015), pRSET_A_-Redoxfluor-C-probe (Yano et al. 2010) and pSY006 (Ohsawa et al. 2017) were used in previous studies.

**Table 2.** Primers used in this study.

Designation	DNA sequence	Resulting plasmids
pHluorin-infusion_Fw	ATCAATGAAACTAGTAGTAAAGGAGAAGAACTTTTCACTG	pMN001
pHluorin-infusion_Rv	ATGTCTAAGAAGCTTTTATTTGTATAGTTCATCCATGCCATG	pMN001
Pex11-pHluorin-infusion_Fw	GACGGCCAGTGAATTCCAAACTTAACCGACCGTTCTTCCATCC	pMN002
Pex11-pHluorin-infusion_Rv	CTCCTTTACTACTAGTTTCATTGATGTATTTTCCCTGCCAGACATCC	pMN002
P11-pH_inv_linker_fw	CAAGTCACCAGAGGAAGACCATGGGTCTTTGTATAGTTCATCCATGCCATGTGTAAT	pMN002
P11-pH_inv_linker_rv	AAGCTTCTTAGACATGACTGTTCCTC	pMN002
mC_inv_linker_fw	TCCTCTGGTGACTTGGTGAGCAAGGGCGAGGAG	pMN003
mC_inv_linker_rv	ATGTCTAAGAAGCTTTTAGGATCTGAGTCCGGACTTGTACA	pMN003
ATG14UP_Fw1	GACGGCCAGTGAATTCTTGGTCAATAAGTGGAAACAGTTTAGCTATGC	pTN001
ATG14DOWN_Rv1	CATGTCTAAGAAGCTTATCTTCTGACACAGGGATTTTCAAATTGTCC	pTN001
ATG14_HIS_Fw	TTTAATTTGCAAGCTGACAGAATTGGGGCTGATTCCAATATTTTGAAG	pTN001
ATG14_HIS_Rv	AAGCTATGGTGTGTGCCCTAAAGTAAGGATCGTATAGGAGTAATCACATATTCC	pTN001
Zeo_Fw	CACACACCATAGCTTCAAAATGTTTCTACTCC	pTN001
Zeo_Rv	AGCTTGCAAATTAAAGCCTTCGAGCG	pTN001
ATG14d_Fw	TGGTCAATAAGTGGAAACAGTTTAGCTATGC	pTN001
ATG14d_Fw	TATCTTCTGACACAGGGATTTTCAAATTGTCC	pTN001
ATG14UP_Fw2	CCGGGGATCCACTAGTGACTTTGGTCAATAAGTGGAAACAG	pARM001
ATG14DOWN_Rv2	TGCCTGCAGCTCGAGCTATTGAAGTTCAACTATCTCCCATTTTTCA	pARM001
pIb1-Arg_Fw	CTCGAGCTGCAGGCA	pARM001
pIb1-Arg_Rv	ACTAGTGGATCCCCGGG	pARM001
CeruleanORF_Fw	ATAGTTGAACTTCAAATGGTGAGCAAGGGCG	pARM002
CeruleanORF_Rv	TGCCTGCAGCTCGAGTTACTTGTACAGCTCGTCCATGC	pARM002
pIb1-Arg_ATG14_Fw	CTCGAGCTGCAGGCAT	pARM002
pIb1-Arg_ATG14_Rv	TTGAAGTTCAACTATCTCCCATTTTTCA	pARM002
ATG14-FLAG_Fw	GACGATGACAAGTAGCTCGAGCTGCAGGCATG	pARM003
ATG14-FLAG_Rv	GTCCTTGTAGTCTTGAAGTTCAACTATCTCCCATTTTTCAT	pARM003
ATG14∆C84-FLAG_Fw	GACTACAAGGACGACGATGACAA	pARM004
ATG14∆C84-FLAG_Rv	ATACTTGTCTGTCATATCATCAATAACTTTTAATTG	pARM004
YFP_KpnI_Fw	GACGGTACCCAAGCTTGTGA	pKS0001
YFP_BamHI_Rv	TCTGGATCCCTAAGAAGCTTTTACT	pKS0001
ATG5_infusion_Fw	CGGGGATCCACTAGTGCTCAAGAGATAAGGAAGTTGAATG	pARM005
ATG5_infusionRv	GCCCTTGCTCACCATAATTATGTATAGCGGTTTGGGCTG	pARM005
ATG6_infusion_Fw	ACGGCCAGTGAATTCGCTACTCAAGGTATCAAACCACTATC	pARM006
ATG6_infusionRv	GGATCCCCGGGTACCCGTTGAACTACTGAAGGCAATTATC	pARM006
pREMIZ_plus_f3	CGCGCAGAAAAAAAGGATCTCAAG	
pREMIZ_plus_r4	CGGAGTCCGAGAAAATCTGG	

**Table 3.** Plasmids used in this study.

Designation	Description	References
pREMI-Z	Introduction of REMI mutagenesis	Mukaiyama et al. (2002)
pIB1	*KpHIS4*	Sears et al. (1998)
pNT204	pIB1 *KpARG4*	Tamura et al. (2010)
SK + Zeo^r^	Zeo^r^	Yano et al. (2010)
PSAP115	*P_KpATG8_-YFP-KpATG8 KpARG4*	Mukaiyama et al. (2004)
pNT812	*P_KpATG8_-YFP-KpATG8 KpHIS4*	Tamura et al. (2010)
pYA006	*P_KpAOX1_-CFP-SKL KpHIS4*	Ano et al. (2005)
pMO500	*P_KpPGK1_-KpPGK1-CFP KpHIS4*	Pang et al. (2019)
pBW1679	*pFA6a-pHluorin-natMX4*	Prosser et al. (2010)
pmCherry-C1	*pmCherry-C1*	Maeda et al. (2015)
pRSET_A_-Redoxfluor-C-probe	*pRSET_A_-Redoxfluor-C-probe*	Yano et al. (2010)
pSY006	pIB1 *3xHA KpHIS4*	Ohsawa et al. (2017)
pMN001	pIB1 *pHluorin*	This study
pMN002	*P_KpPex11_-KpPEX11-pHluorin KpHIS4*	This study
pMN003	*P _KpPex11_-KpPEX11-pHluorin-mCherry KpHIS4*	This study
pTN001	*P_KpATG14_-KpATG14-T_KpATG14_ KpHIS4*	This study
pTN002	*KpATG14∆::Zeo^r^*	This study
pARM001	*P_KpATG14_-KpATG14 KpARG4*	This study
pARM002	*P_KpATG14_-KpATG14-Cerulean KpARG4*	This study
pARM003	*P_KpATG14_-KpAtg14-FLAG KpARG4*	This study
pARM004	*P_KpATG14_-KpATG14∆C84-FLAG KpARG4*	This study
pKS0001	*P_KpIDH1_-KpIDH1-YFP KpARG4*	This study
pARM005	*P_KpATG5_-KpATG5-Cerulean KpARG4*	This study
pARM006	*P_KpATG6_-KpATG6-3xHA KpHIS4*	This study

The vector used for FACS-based screening was constructed as follows: The primer pair pHluorin-infusion_Fw/pHluorin-infusion_Rv was used to amplify the fluorescent protein pHluorin-coding sequence using pBW1679 as template. The PCR-amplified fragment was then fused with *Spe*I/*Hind*III cut fragment of pIB1, using In-Fusion Cloning Kit (Clontech), which resulted in pMN001. Then, the KpPex11 promoter and the ORF region without the STOP codon were amplified using primers Pex11-pHluorin-infusion_Fw/Pex11-pHluorin-infusion_Rv using the genomic DNA as template. The obtained fragment was fused with *Eco*RI/*Spe*I cut fragment of pMN001 using In-Fusion Cloning Kit (Clontech), which resulted in pMN002. Finally, the primer pairs P11-pH_inv_linker_fw/P11-pH_inv_linker_rv and mC_inv_linker_fw/mC_inv_linker_rv were used to linearize pMN002 and amplify the fluorescent protein mCherry-coding sequence from pmCherry-C1 as a template, respectively. These fragments were fused with each other, resulting in the vector pMN003 used for FACS-based screening.

A deletion cassette for the *KpATG14* gene was constructed as follows: Primer pair ATG14UP_Fw1/ATG14DOWN_Rv1 was used to amplify a 3.1-kb fragment using the genomic DNA as template. The fragment, including *KpATG14* ORF and its upstream and downstream regions, was cloned into pIB1 between *Eco*RI and *Hind*III sites using In-Fusion Cloning Kit (Clontech), resulting in pTN001. Then, *KpATG14* ORF, amplified by the primer pair ATG14_HIS_Fw/ATG14_HIS_Rv, was replaced by the zeocin resistance gene that was amplified from SK + Zeo^r^ using the primer pair Zeo_Fw/Zeo_Rv, yielding the *KpATG14* disruption vector pTN002. To disrupt the *KpATG14* gene, the disruption cassette was PCR-amplified from the disruption vector, pTN001, using the primer pair ATG14d_Fw/ATG14d_Fw, and transformed into *K. phaffii* PPY12 by electroporation. Proper gene disruptions were confirmed by colony PCR.

The vectors harboring tagged *KpATG14* were constructed as follows: the *KpATG14* promoter and ORF region were PCR-amplified using primers ATG14UP_Fw2 and ATG14DOWN_Rv2 and using the genomic DNA as template. The fragment was fused to pIB1, which was linearized by PCR with the primers pIb1-Arg_Fw and pIb1-Arg_Rv, resulting in pARM001. The fluorescence protein Cerulean-coding sequence was amplified from pRSET_A_-Redoxfluor-C-probe as a template with primers CeruleanORF_Fw/CeruleanORF_Rv and inserted into pARM001, which was linearized by PCR with the primers pIb1-Arg_ATG14_Fw/pIb1-Arg_ATG14_Rv, resulting in pARM002. The infusion reaction to construct these vectors was performed with In-Fusion Cloning Kit (Clontech). For the construction of pARM003 and pARM004, pARM001 was linearized by PCR with the primer pairs ATG14-FLAG_Fw/ATG14-FLAG_Rv and ATG14-FLAG∆C84_F/ATG14∆C84-FLAG_Rv, respectively, and the PCR products were subjected to self-ligation.

The vector encoding *KpIDH1-YFP* expressed under its native promoter was constructed by swapping *GFP* coding sequence with *YFP* coding sequence. The vector pIB1-IDH1-GFP, used previously (Yamashita et al. 2016), was linearized by restriction enzymes *Kpn*I and *Bam*HI. The fragment *YFP* coding sequence was PCR amplified with a primer set YFP_KpnI_Fw/YFP_BamHI_Rev using pNT812 as a template, and treated with restriction enzymes *Kpn*I and *Bam*HI. These two fragments were ligated with Ligation-Convenience Kit (NIPPON GENE), resulting in pKS0001.The vector encoding *KpATG5-CERULEAN* expressed under its native promoter was constructed as follows: the *KpATG5* promoter and ORF region without the STOP codon were amplified using primers ATG5_infusion_Fw/ATG5_infusion_Rv using the genomic DNA as template. The obtained fragment was inserted into pARM002, which was linearized by PCR with primers pIb1-Arg_Rv/ATG14∆C84-Ceruean_Fw, resulting in pARM005. The vector encoding *KpATG6-3xHA* expressed under its native promoter was constructed as follows: The *KpATG6* promoter and ORF region without the STOP codon were amplified using primers ATG6_infusion_Fw/ATG6_infusion_Rv using the genomic DNA as template. The obtained fragment was inserted into pSY006, which was linearized by the enzyme cut with *Kpn*I and *Eco*RI, resulting in pARM006. The infusion reactions were performed using In-Fusion Cloning Kit (Clontech).

### Restriction enzyme-mediated integration mutagenesis

Restriction enzyme-mediated integration (REMI) was used to facilitate a random mutation of genes in the *K. phaffii* genome by the incorporation of a zeocin-resistance gene. Transformation of *K. phaffii* with a pREMI-Z vector was carried out after linearizing it with *Bam*HI. Transformed cells were selected by growth on zeocin plates. Those transformed cells unable to degrade peroxisomes during ethanol adaptation were identified through the two-step (FACS and fluorescence microscopy) screening, described below. Basic methods used for the identification of the genes required for macropexophagy were described previously (Schroder et al. 2007). To find mutation sites, sequencing analyses were performed with the primer set pREMIZ_plus_f3 and pREMIZ_plus_r4.

### Pexophagy induction

Macropexophagy for the FACS based screening was induced as follows. A single colony was inoculated onto YPD medium and cultivated overnight. A volume of 40 µl of cells in 5 ml of YPD were transferred to 5 ml of fresh YPD medium and incubated for 5 h as a preculture. To prepare the cell samples used for the first screening with FACS, precultured cells were grown in 5 ml of SM medium for 12–16 h, transferred to 5 ml of SE medium and incubated for 6 h to induce macropexophagy. For the second screening with fluorescence microscope, cells plated on a membrane (Amersham HybondTM-N+, GE Healthcare) were incubated on SM agar medium for 18 h. Then, the membrane was transferred onto SE agar medium and incubated for 4 h to induce macropexophagy. Macropexophagy and micropexophagy in investigating the function of KpAtg14 were induced as follows. A single colony was inoculated onto YPD medium and cultivated overnight. The cells were transferred to 5 ml of YNM medium containing 0.93 µg/ml FM4-64 (Invitrogen), which labeled the vacuolar membrane. The cells were incubated in 5 ml of YNM medium for 12–16 h and were shifted to 5 ml of YNE medium (macropexophagy) or YND medium (micropexophagy) and examined.

### Bulk autophagy and mitophagy induction

A single colony was inoculated into YPD medium and cultivated overnight. A volume of 40 µl of cells in 5 ml of YPD were transferred to 5 ml of fresh YPD medium and incubated for 5 h as a preculture. Then, the precultured cells were transferred to SD-N medium.

### FACS

**Figure 1.**
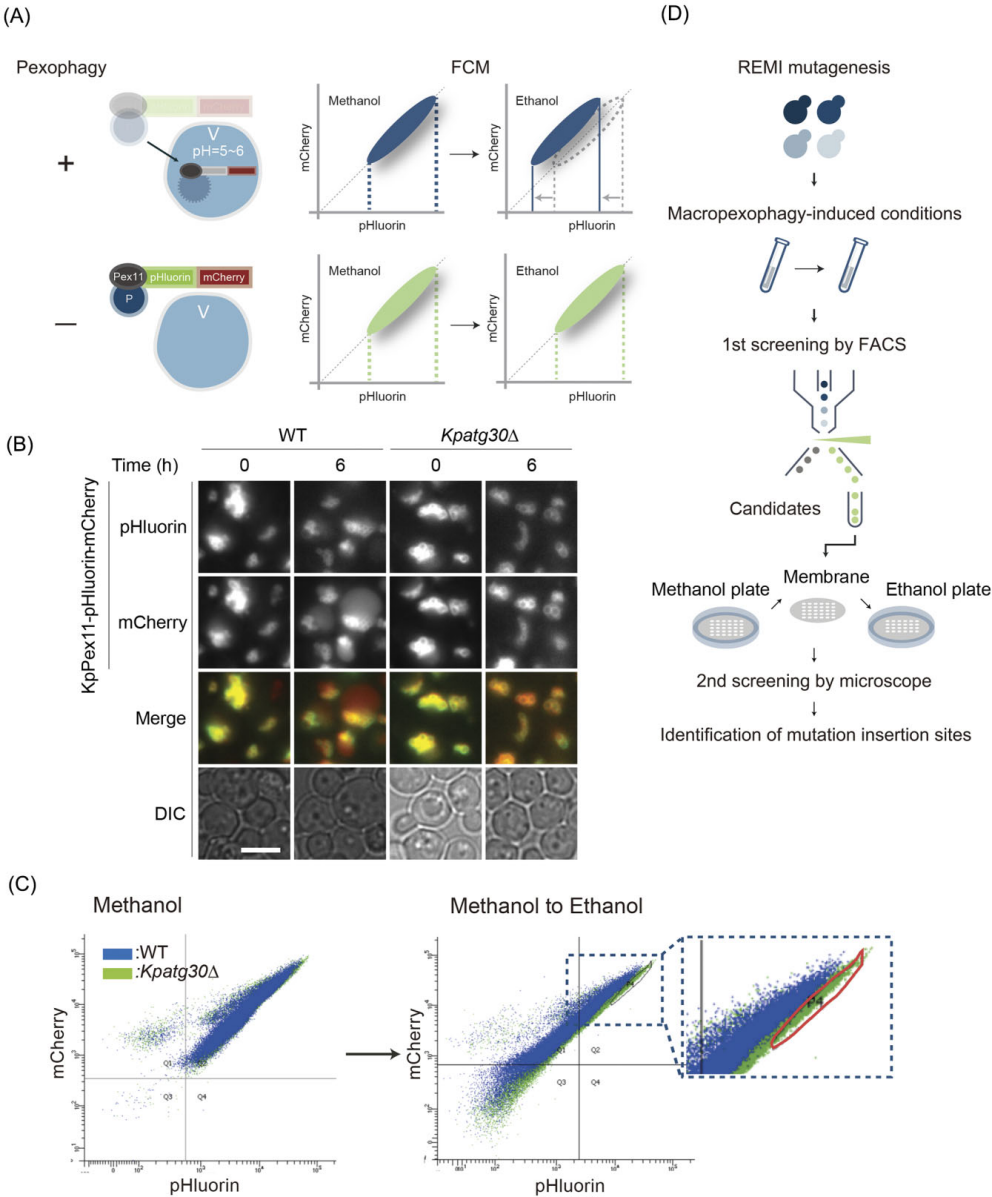
Establishment of the FACS-based screening system for macropexophagy mutants. (A) Conceptual diagram of the methodology adopted in the screening method for discovering macropexophagy mutants using the KpPex11-pHluorin-mCherry reporter. V, vacuole; P, peroxisome; +, pexophagy active; −, pexophagy defective; and FCM, flow cytometry. (B) Microscopic images of WT and *Kpatg30Δ* cells expressing KpPex11-pHluorin-mCherry. Cells were observed 6 h after the carbon-source shift from methanol to ethanol. Merged images are combined images of pHluorin and mCherry fluorescence images. Bar; 2 µm. (C) Representative flow cytometry plot for sorting WT and *atg30Δ* cells expressing KpPex11-pHluorin-mCherry. The left panel shows the population of cells grown on methanol, whereas the right panel displays the population of cells transferred from a methanol to an ethanol-containing medium and incubated for 6 h. Scater plots of WT cells (blue) are overlaid with those of *Kpatg30Δ* cells (green). The sorting gate for screening macropexophagy mutants is indicated by the circle (red). (D) Schematic overview of the experimental protocol. After REMI mutagenesis of WT cells expressing KpPex11-pHluorin-mCherry was introduced by transforming the pREMI-Z vector, all the transformants were collected for the first screening by FACS. The sorted candidates were further evaluated by fluorescence microscopy as the second screening. Finally, sequencing analysis was conducted to identify the mutation insertion sites and the genes required for macropexophagy.

Cells were resuspended in ice-cold phosphate-buffered saline buffer and kept on ice. Flow cytometry was performed using FACSAria III (Becton Dickinson). Fluorescent channel and light scatter were set at log gain. The forward scatter (FSC) was set at a photomultiplier tube (PMT) voltage of 23 with a threshold of 800. The PMT voltages of side scatter (SSC) and green fluorescent protein (GFP) were set at 225 and 500, respectively. pHluorin fluorescence was excited with a 488-nm laser, and the emission at 530/30 nm was detected. mCherry fluorescence was excited with a 561-nm laser, and the emission at 610/20 nm was detected. At least 50 000 cells were analyzed per sample. Theoretically, *KpPEX11-*promoter-dependent fluorescence of pHluorin and mCherry is detected when cells are grown on methanol, showing the correlated dot plots of the cell population both in pexophagy-active and -defective cells. After the carbon source shifts to ethanol, the fluorescence of pHluorin is decreased in pexophagy-active cells, while the fluorescence of mCherry is maintained. On the other hand, the fluorescence of both pHluorin and mCherry are maintained in pexophagy-defective cells. Therefore, when the dot plots of pexophagy-active and -defective cells are merged, pexophagy-active cell populations can be observed. As such, cells were gated for FSC and SSC to select single cells, as described in Fig. 1(C). FACSDiva 8 software (Becton Dickinson) was used for data acquisition and creation of scatter plots. Bioconductor (www.bioconductor.org) on the open-source statistical platform R (www.r-project.org) was used for processing data, followed by the preparation of histograms. Candidates of pexophagy mutants were sorted and transferred into 5 ml of YPD medium. After harvest, cells were plated onto YPD agar medium and incubated for 2–3 days until colonies appeared. These colonies, candidates of pexophagy mutants, were used for the second screening with fluorescence microscopy.

### Fluorescence microscopy

Cells were observed using an IX81 fluorescence microscope (Olympus) equipped with a XF52 filter set (Omega Optical Inc.) for FM4-64 and fluorescent proteins. Image data were captured with a SenSysTM charged-coupled device camera (PhotoMetrics) and analyzed on MetaMorph imaging software (Universal Imaging Corporation), ImageJ2 version 2.3.0 and Adobe Illustrator. In all figures, the scale bar was set to 2 µm.

### Morphometric analysis

Cell count analysis was performed in cells observed under fluorescence microscopy (*n* > 30, *n*; number of cells analyzed and *f* > 3, *f*; a field of vision). Independent examinations were repeated at least three times.

### Preparation of protein extracts from yeast cells

The samples were prepared from cells harvested at an OD_610_ of 2.0. The cells were resuspended in 1 ml of solution I that contained 0.2 M NaOH and 0.5% (v/v) 2-mercaptoethanol. Then the cell samples were incubated on ice for 10 min and 0.1 ml of 100% (w/v) trichloroacetic acid solution was added. The cell lysates were subjected to centrifugation at 20 000 g at 4°C for 5 min. After the supernatant was removed, the pellet was resuspended in 1 ml of acetone by brief sonication. The obtained sample was then subjected to centrifugation at 20 000 *g* at 4°C for 5 min and the pellets were dried. Subsequently, the pellets were dissolved in 80 µl of sample buffer, containing 0.1 M Tris-HCl (pH 7.5), 2% (w/v) SDS, 1% (v/v) glycerol, 0.5% (v/v) 2-mercaptoethanol, and 0.01% (w/v) bromophenol blue, by brief sonication. The samples were then incubated at 65°C for 10 min, followed by centrifugation at 20 000 g for 1 min. A 20-µl volume of the supernatant was electrophoresed on a 10% SDS–PAGE gel.

### Immunoblot analysis

Proteins were transferred to a PVDF membrane using semidry blotting (ATTO, Tokyo, Japan). After incubation in Bloking One (Nacalai tesque) for 1 h at room temperature, the membranes were incubated overnight with anti-GFP antibody (JL-8; Clontech), anti-FLAG antibody (M2; Merck Millipore, Darmstadt, Germany), anti-Pgk1 antibody (22C5D8; Invitrogen, Waltham, MA, USA) or anti-β-actin (ab8224; Abcam, Cambridge, UK) at a 1:1000 dilution in TBS-T buffer, which was composed of 20 mM Tris, 0.1% (v/v) Tween 20, 0.8% (w/v) NaCl, and 0.02% (w/v) KCl. The membranes were washed three times with TBS-T buffer and incubated with HRP-conjugated antimouse IgG (12–349; Merck Millipore) at a 1:10 000 dilution for 1 h. Finally, bound secondary antibodies were detected using Western Lightning (Perkin-Elmer Life Science, Waltham, MA, USA) and the signals were analyzed using a Light Capture system (ATTO).

### Amino acid sequence analyses

To identify KpAtg14 orthologs in other methylotrophic yeasts, Position-Specific Iterated BLAST (PSI-BLAST) searches were performed on the National Center for Biotechnology Information (NCBI) library (http://www.ncbi.nlm.nih.gov/BLAST/blast.cgi), using the coding regions of *K. phaffii* accession GQ67_04301T0 (AOA63598.1) as queries. The KpAtg14 protein sequences were aligned with Clustal Omega (https://www.ebi.ac.uk/Tools/msa/clustalo/) for multiple sequence alignment and EMBOSS Needle (https://www.ebi.ac.uk/Tools/psa/emboss_needle/) was used for pairwise sequence alignment under default settings. The web-based tool, Waggawagga, was used for the comparative visualization of coiled-coil predictions (https://bio.tools/waggawagga).

### Structural analysis using AlphaFold2

AlphaFold2-multimer (Evans et al. 2022) was used through a subscription to the Google Colab (https://colab.research.google.com/github/sokrypton/ColabFold/blob/main/AlphaFold2.ipynb#scrollTo=svaADwocVdwl) following guidelines on the document (Jumper et al. 2021). Superposition of AlphaFold2 predictions on known structures was performed using the align command in PyMOL (The PyMOL Molecular Graphics System, Version 2.3.5 Schrödinger, LLC).

### Immunoprecipitation

*Komagataella phaffii* cells were collected, resuspended in HNE buffer (25 mM HEPES-KOH pH 7.2, 150 mM NaCl, and 2 mM EDTA), and ruptured using Multi-Beads Shocker (Yasui Kikai) at 2500 rpm for 30 s with 0.5-mm YZB zirconia beads (Yasui Kikai). Subsequently, the same volume of HNE buffer containing 0.2% dodecyl-β-D-maltoside (DDM, from Dojindo Molecular Technologies) was added to the lysates, incubated for 20 min on ice, and then centrifuged at 10 000 rpm for 15 min. The supernatants were incubated with anti-FLAG magnetic beads (Wako Pure Chemical) with gentle rotation for 3 h. The beads were then washed three times with the HNE buffer containing 0.03% DDM and the bound proteins were eluted with SDS–PAGE sample buffer.

## Results

### The FACS-based screening identified the genes required for macropexophagy

The yeast cells, expressing a peroxisomal protein KpPex11 (Oku and Sakai 2016) fused with a tandem-tagged fluorescent protein pHluorin-mCherry, were used for FACS-based screening. In principle, when KpPex11-pHluorin-mCherry encounters the vacuole via the pexophagic process, the fluorescence signal of the pH-sensitive GFP variant pHluorin is reduced under the internal low pH conditions, while mCherry exhibited diffused fluorescence, which could be sorted through the screening (Fig. 1A). To examine the methodological validity, we first observed the wild-type (WT) strain and *Kpatg30∆* strain, a complete pexophagy-defective mutant (Farré et al. 2008, Ohsawa et al. 2021), expressing KpPex11-pHluorin-mCherry by a fluorescence microscope during macropexophagy-induced conditions. As expected, upon the induction of macropexophagy, reduction of pHluorin fluorescence and diffusion of mCherry fluorescence were observed in the WT strain, whereas in the *Kpatg30∆* strain both pHlluorin and mCherry fluorescence were clearly detected under macropexophagy-induced conditions (Fig. 1B). Next, we analyzed the WT and *Kpatg30∆* strains by flow cytometry during the shift of carbon source from methanol to ethanol, inducing macropexophagy. When these strains were grown on methanol, an identical plot pattern of cell populations was obtained, with the fluorescence intensity of pHlluorin and mCherry showing a correlation (Fig. 1C). After the shift to ethanol-containing medium, on the other hand, the WT strain showed more cell populations emitting weaker fluorescence intensity of pHlluorin than the *Kpatg30∆* strain. In particular, in the area indicated by the red circle in the enlarged image, only four plots appeared in the WT strain, whereas 5764 plots were present in the *Kpatg30*∆ strain (Fig. 1C), suggesting that the flow cytometry analysis can distinguish WT cells from *Kpatg30*∆ cells and that the highlighted area can be used as a sorting gate for screening macropexophagy mutants.

Using the WT strain expressing KpPex11-pHluorin-mCherry, we designed an experimental protocol to identify the genes required for macropexophagy, which entailed a two-step screening processes by FACS and fluorescence microscopy (Fig. 1D). Accordingly, random mutagenesis was performed and ~50 000 transformants were obtained. Transformants were then screened using FACS by selecting the cells sorted by the gate shown in Fig. 1(C), which yielded 227 candidates for the second screening (data not shown). The candidate transformants were then spotted on a membrane, which was placed on a methanol-containing medium and transferred to an ethanol-containing medium, inducing macropexophagy. Fluorescence microscopy analysis narrowed the list of candidates down to 84 (data not shown). Subsequently, sequencing analysis was performed to find mutation sites in the 84 transformants and 9 different genes that had mutations in their ORF region were identified (Table 4). Among the nine genes, we found one unknown gene that contained a 1584-bp ORF encoding a protein of 528 amino acids. The other eight genes, namely *ATG1, ATG7, ATG9, ATG11, ATG12, ATG18, ATG24*, and *ATG26* (Yuan et al. 1999, Kim et al. 2001, Strømhaug et al. 2001, Mukaiyama et al. 2002, 2004, Oku et al. 2003, Stasyk et al. 2003, Ano et al. 2005), have already been discovered and characterized by previous studies. A PSI-BLAST search (Jones and Swindells 2002) at the NCBI database found that the amino acid sequence of the unknown gene had a weak but significant homology to ScAtg14 in *S. cerevisiae* (Identity: 14%, Similarity: 27%) (Fig. S1). Therefore, we named the putative gene *KpATG14* and performed the following experiments.

**Table 4.** List of *ATGs* identified in this study.

Gene	Description	References
*ATG1*	Serine/threonine-kinase	Strømhaug et al. (2001), Mukaiyama et al. (2002)
*ATG7*	E1 (ubiquitin-activating enzyme)-like function	Yuan et al. (1999), Mukaiyama et al. (2004)
*ATG9*	Integral membrane protein	Strømhaug et al. (2001), Mukaiyama et al. (2002)
*ATG11*	Coiled-coil domain	Kim et al. (2001)
*ATG12*	Component of Atg5/Atg12/Atg16 complex	Mukaiyama et al. (2002)
*ATG14*		This study
*ATG18*	Protein with WD40 motifs	Guan et al. (2001)
*ATG24*	Sorting nexin with PX domain	Ano et al. (2005)
*ATG26*	UDP-glucose:sterol glucosyl-transferase	Oku et al. (2003), Stasyk et al. (2003)

### Characterization of KpAtg14

**Figure 2.**
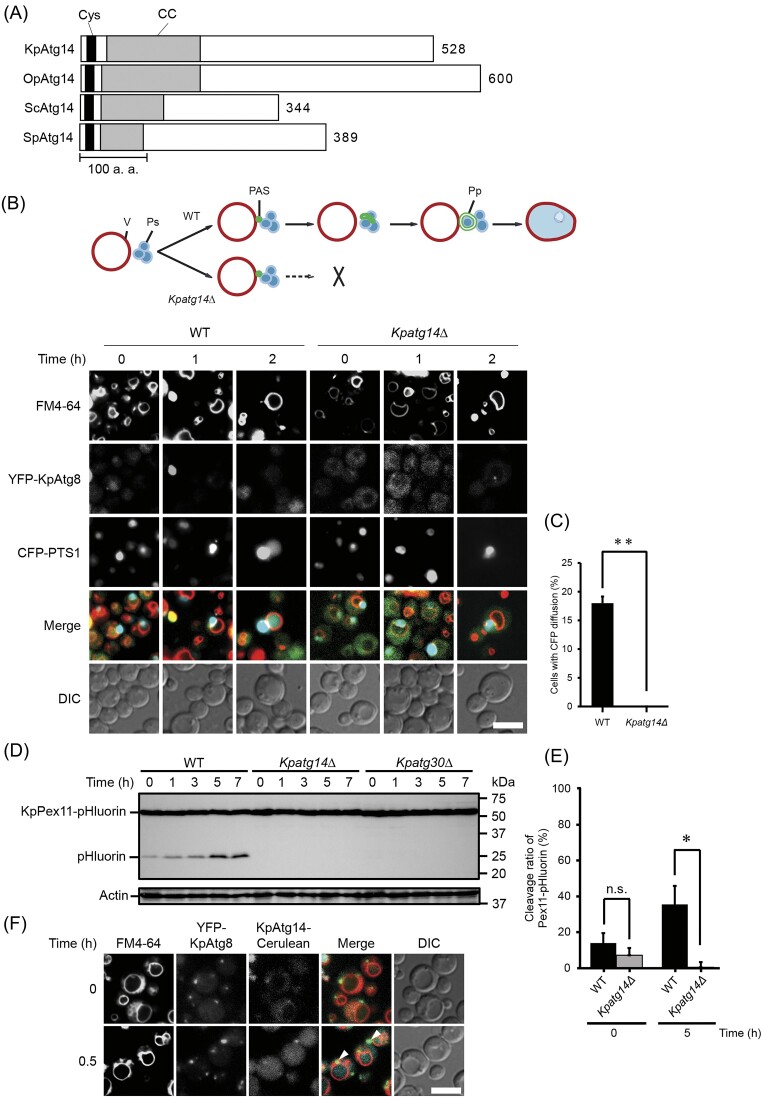
Identification of the requirement of KpAtg14 for macropexophagy. (A) Structural comparison of Atg14 proteins from *K. phaffii, O. polymorpha, S. cerevisiae*, and *S. pombe*. Cys, cysteine repeat domain; CC, coiled-coil domain. (B) Schematic image and fluorescent microscopy analysis of the WT and *Kpatg14Δ* strains expressing YFP-KpAtg8 and CFP-PTS1 during macropexophagy. The vacuolar membranes were stained with FM4-64. Merged images are combined images of FM4-64, YFP-KpAtg8, and CFP-PTS1 images. DIC, differential interference contrast microscopy. Bar; 2 µm. For the schematic image, V, Ps, PAS, and Pp indicate vacuole, peroxisomes, preautophagosomal structure, and pexophagosome, respectively. (C) Quantification of the cells with CFP diffusion in the WT and *Kpatg14Δ* strains. Microscopic images of the cells 2 h after macropexophagy induction (see Fig. 2B) were used for analysis. Quantified values are shown as the means ± standard error (SE) of three independent experiments. **P < .01 versus control. (D) Immunoblot analysis of pHluorin-tagged KpPex11 in *K. phaffii* WT, *Kpatg14Δ* and *Kpatg30Δ* cells under macropexophagy-induced conditions. (E) Quantification of the intensity of (pHluorin)/(pHluorin + KpPex11-pHluorin) in the WT and *Kpatg14Δ* strains. The bands detected 0 and 5 h after macropexophagy induction (see Fig. 2D) were used for analysis. Quantified values are shown as the means ± SE of three independent experiments. *P < .05 versus control. (F) Fluorescent microscopy analysis of the WT strain expressing YFP-KpAtg8 and KpAtg14-Cerulean during macropexophagy. The vacuolar membranes were stained with FM4-64. Merged images are combined images of FM4-64, YFP-KpAtg8, and KpAtg14-Cerulean images. DIC, differential interference contrast microscopy. Bar; 2 µm. Arrows indicate the colocalization of YFP-Atg8 and Atg14-Cerulean.

We first asked whether KpAtg14 contained conserved amino acid sequences found among yeasts. The PSI-BLAST search with KpAtg14 identified a putative OpAtg14 in another methylotrophic yeast *Ogataella polymorpha* together with well-characterized ScAtg14 in *S. cerevisiae* and SpAtg14 in *S. pombe*. Alignment analysis of KpAtg14 showed relatively low homologies to SpAtg14 (identity: 13%, similarity: 39%) and OpAtg14 (identity: 18%, similarity: 54%), similar to ScAtg14 (Fig. S1). Investigating the amino acid sequences, we found that KpAtg14, as well as OpAtg14, contained a conserved sequence motif of cysteine repeat at the N terminal region (Fig. S1). In addition, the coiled-coil prediction (Simm et al. 2015) indicated that KpAtg14 and OpAtg14 possessed a conserved sequence motif of a coiled-coil domain at the N terminal region (Fig. S2). These results indicated that KpAtg14 has conserved motifs found among yeasts (Fig. 2A).

### Functional analysis shows that KpAtg14 is required for macropexophagy

The *KpATG14* gene was disrupted by replacing the ORF with the zeocin resistance gene as a selective marker. We then investigated the role of KpAtg14 in macropexophagy by performing the morphological analysis in the *Kpatg14Δ* strain. A fluorescent protein CFP fused with the peroxisomal targeting signal 1 (PTS1) and YFP-tagged KpAtg8 were used as markers of peroxisomes and pexophagosomes, respectively (Mukaiyama et al. 2004). The vacuolar membranes were stained with FM4–64. YFP-KpAtg8 was observed as dot-like structures both in the WT and *Kpatg14Δ* strains (Fig. 2B). However, clear CFP diffusion in the vacuole was observed only in the WT strain 2 h after the medium shift (Fig. 2B and C). In the *Kpatg14Δ* strain, CFP fluorescence remained by the side of the vacuole, suggesting that the *Kpatg14Δ* strain is a complete macropexophagy-defective mutant. Macropexophagic activity was also investigated by immunoblot analysis using cells expressing KpPex11 fused with a fluorescent protein pHluorin, as described previously (Yamashita et al. 2017). Peroxisome degradation was confirmed by detecting the cleavage of KpPex11-pHluorin in the WT strain. On the other hand, the cleaved form of pHluorin was detected in neither the *Kpatg14Δ* nor the *Kpatg30Δ* strain (Fig. 2D and E), which was consistent with the microscopic analyses. Furthermore, we examined whether Cerulean-tagged KpAtg14 colocalizes with YFP-KpAtg8 under macropexophagy-induced conditions. Upon the induction of macropexophagy, KpAtg14-Cerulean was observed to colocalize with YFP-KpAtg8 (Fig. 2F). Taken together, we concluded that KpAtg14 was essential for macropexophagy in *K. phaffii*.

### KpAtg14 is also required for micropexophagy

**Figure 3.**
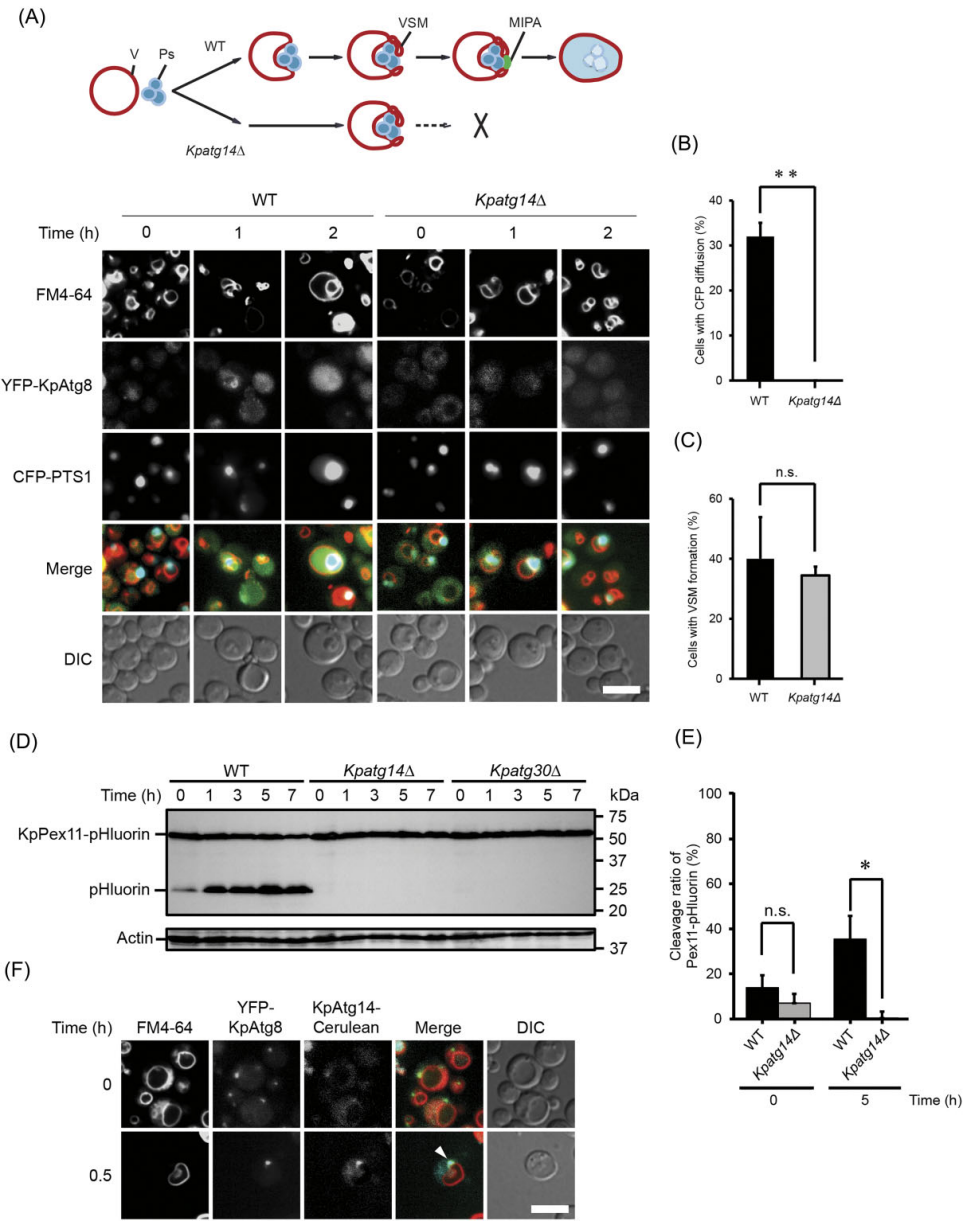
Functional analysis of KpAtg14 under micropexophagy-induced conditions. (A) Schematic image and fluorescent microscopy analysis of the WT and *Kpatg14Δ* strains expressing YFP-KpAtg8 and CFP-PTS1 during micropexophagy. The vacuolar membranes were stained with FM4-64. Merged images are combined images of FM4-64, YFP-KpAtg8, and CFP-PTS1 images. DIC, differential interference contrast microscopy. Bar; 2 µm. For the schematic image, V, Ps, VMS, and MIPA indicate vacuole, peroxisomes, VSMs, and micropexophagic membrane apparatus, respectively. (B) Quantification of the cells with CFP diffusion in the WT and *Kpatg14Δ* strains. Microscopic images of the cells 2 h after micropexophagy induction (see Fig. 3A) were used for analysis. Quantified values are shown as the means ± SE of three independent experiments. **P < .01 versus control. (C) Morphometric analysis of VSM formation. Quantified values are shown as the means ± SE of three independent experiments. n.s. = not significant. (D) Immunoblot analysis of pHluorin-tagged KpPex11 in *K. phaffii* WT, *Kpatg14Δ*, and *Kpatg30Δ* cells under micropexophagy-induced conditions. (E) Quantification of the intensity of (pHluorin)/(pHluorin + KpPex11-pHluorin) in the WT and *Kpatg14Δ* strains. The bands detected 0 and 5 h after micropexophagy induction (see Fig. 3D) were used for analysis. Quantified values are shown as the means ± SE of three independent experiments. n.s. = not significant and *P < .05 versus control. (F) Fluorescent microscopy analysis of the WT strain expressing YFP-KpAtg8 and KpAtg14-Cerulean during micropexophagy. The vacuolar membranes were stained with FM4-64. Merged images are combined images of FM4-64, YFP-KpAtg8, and KpAtg14-Cerulean images. DIC, differential interference contrast microscopy. Bar; 2 µm. Arrows indicate the colocalization of YFP-KpAtg8 and KpAtg14-Cerulean.

Next, we asked whether KpAtg14 is essential for micropexophagy. Morphological analysis was performed in the WT and *Kpatg14Δ* strains, in a manner similar to the macropexophagic observation described above. In the WT strain, a cluster of peroxisomes started to be enwrapped by the fragmented vacuoles with the association of YFP-KpAtg8 1 h after the medium shift and a diffusion of the CFP fluorescence was detected in the following hours, indicating the occurrence of micropexophagy (Fig. 3A and B). On the other hand, in the *Kpatg14Δ* strain, although peroxisomes were enwrapped by the fragmented vacuoles in a similar manner to the WT strain, CFP diffusion was not observed (Fig. 3A and B). Detailed morphometric analysis with the yeast cells 1 h after the macropexophagy induction confirmed that the same level of VSMs budding from the vacuole was detected in the WT and *Kpatg14Δ* strains (Fig. 3C), suggesting that KpAtg14, together with other ATG proteins, plays an indispensable role in the final stage of micropexophagy such as MIPA formation for the complete enclosure of peroxisomes in the vacuole (Mukaiyama et al. 2002). Our previous study proposed a model of micropexophagy in *K. phaffii*, in which micropexophagy is divided into four distinct morphological stages, termed stage 0 to stage 3 (Sakai et al. 1998b). Considering that peroxisomes were enwrapped by VSM in a similar manner to the WT strain, the phenotype of the deletion of *KpATG14* could be categorized into stage 1c in which most *ATG* mutants such as *Kpatg1Δ* and *Kpatg7Δ* are grouped. Furthermore, immunoblot analysis with the WT, *Kpatg14Δ*, and *Kpatg30Δ* strains found that micropexophagic activity was defective in the *Kpatg14Δ* and *Kpatg30Δ* strains (Fig. 3D and E). We also examined the intracellular localization of KpAtg14-Cerulean and YFP-KpAtg8, a marker protein of MIPA under macropexophagy-induced conditions (Mukaiyama et al. 2004). Upon the induction of micropexophagy, KpAtg14-Cerulean was observed to colocalize with YFP-KpAtg8 (Fig. 3F). These results indicated that KpAtg14 is required for micropexophagy in *K. phaffii*.

### KpAtg14 is dispensable for mitophagy

**Figure 4.**
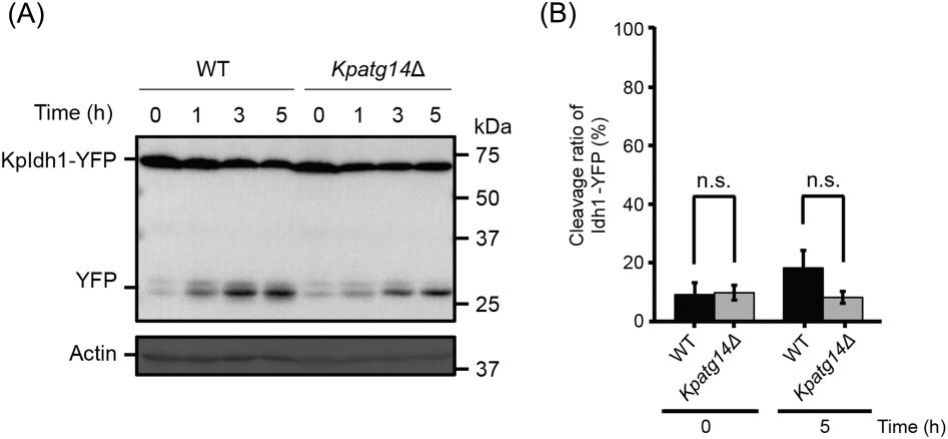
Functional analysis of KpAtg14 under starvation-induced mitophagy conditions. (A) Immunoblot analysis of YFP-tagged KpIdh1 in *K. phaffii* WT and *Kpatg14Δ* cells under nitrogen-starvation conditions. (B) Quantification of the intensity of (YFP)/(YFP + KpIdh1-YFP) in the WT and *Kpatg14Δ* strains. The bands detected 0 and 5 h after mitophagy induction (see Fig. 4A) were used for analysis. Quantified values are shown as the means ± SE of three independent experiments. n.s. = not significant.

Subsequently, we investigated the effects of deletion of *ATG14* on mitophagy, another selective autophagy pathway for mitochondria. To monitor the mitophagic activity, the yeast strains expressing a mitochondrial matrix protein KpIdh1 fused with YFP were constructed (Yamashita et al. 2016). The cells cultured on a nutrient-rich medium were transferred to nitrogen-starvation conditions and collected over time for immunoblot analysis. The cleaved form of YFP was clearly detected in the WT strain during starvation-induced mitophagy (Fig. 4A and B). In the *Kpatg14Δ* strain, mitophagy was slightly impaired but active, indicating that KpAtg14 is not required for starvation-induced mitophagy. Considering that ScAtg14 is indispensable for mitophagy in the budding yeast *S. cerevisiae* (Yamashita et al. 2016), *K. phaffii* may have different regulation mechanism for mitophagy.

### KpAtg14 is required for nonselective bulk autophagy

**Figure 5.**
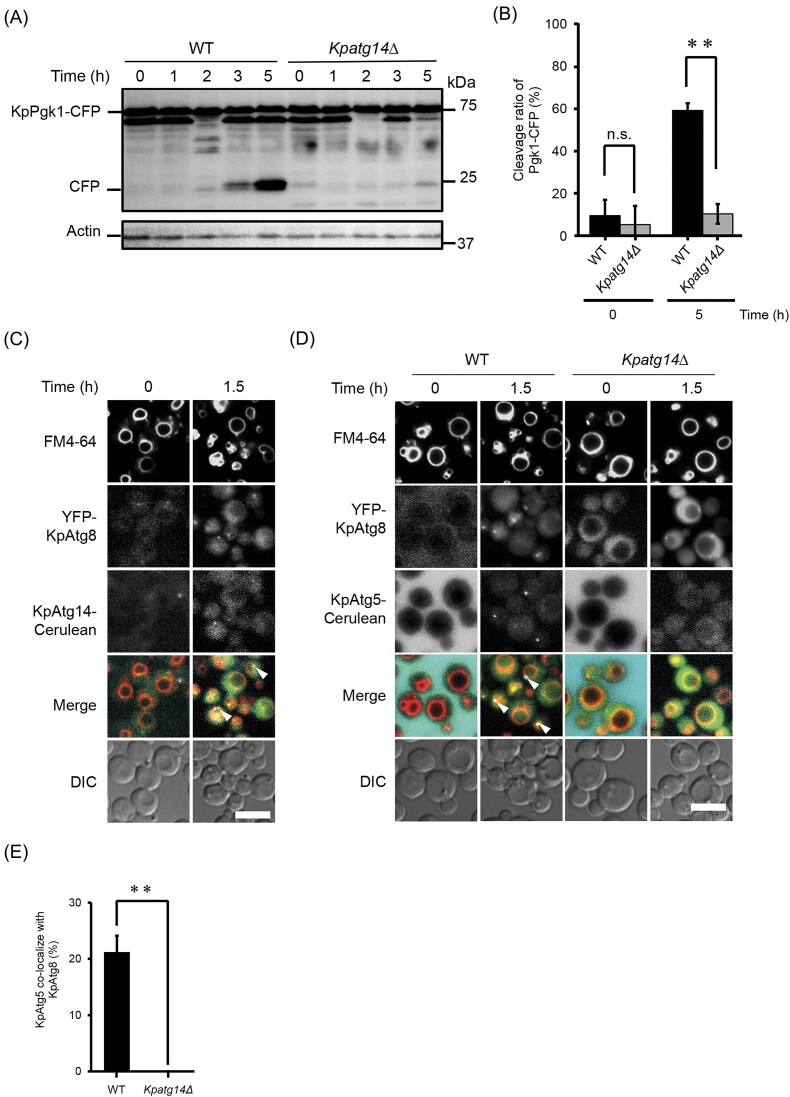
Functional analysis of KpAtg14 under bulk-autophagy-induced conditions. (A) Immunoblot analysis of CFP-tagged KpPgk1 in *K. phaffii* WT and *Kpatg14Δ* cells under bulk autophagy-induced conditions. (B) Quantification of the intensity of (CFP)/(CFP + KpPgk1-CFP) in the WT and *Kpatg14Δ* strains. The bands detected 0 and 5 h after bulk-autophagy induction (see Fig. 5A) were used for analysis. Quantified values are shown as the means ± SE of three independent experiments. n.s. = not significant and **P < .01 versus control. (C) Fluorescent microscopy analysis of the WT strain expressing YFP-KpAtg8 and KpAtg14-Cerulean during bulk autophagy. The vacuolar membranes were stained with FM4-64. Merged images are combined images of FM4-64, YFP-KpAtg8, and KpAtg14-Cerulean images. DIC, differential interference contrast microscopy. Bar; 2 µm. Arrows indicate the colocalization of YFP-KpAtg8 and KpAtg14-Cerulean. (D) Fluorescent microscopy analysis during bulk-autophagy. The vacuoles were stained with FM4-64. To visualize *de novo*-synthesized PAS and downstream factor, YFP-KpAtg8 and KpAtg5-Cerulean were used as their marker. The vacuolar membranes were stained with FM4-64. Merged images are combined images of FM4-64, YFP-KpAtg8 and KpAtg5-Cerulean images. DIC, differential interference contrast microscopy. Bar; 2 µm. Arrows indicate the colocalization of YFP-KpAtg8 and KpAtg5-Cerulean. (E) Ratio of KpAtg5 puncta colocalizing with KpAtg8, estimated from analysis of the fluorescent images shown in Fig. 5(D). For each sample, a minimum of 50 cells were analyzed. Quantified values are shown as the means ± SE of three independent experiments. **P < .01 versus control in each condition.

We then examined whether KpAtg14 is required for nonselective bulk autophagy with the yeast strains expressing the cytoplasmic protein KpPgk1 fused with CFP, as previously described (Pang et al. 2019). When the cells were subjected to nitrogen-starvation conditions, the cleaved form of CFP was clearly detected in the WT strain (Fig. 5A and B). On the other hand, the deletion of the *ATG14* gene severely impeded bulk autophagic degradation under nitrogen starvation. We also investigated the intracellular localization of KpAtg14. When cells were cultured on YPD medium, KpAtg14-Cerulean was diffused in the cytosol. Upon the induction of bulk autophagy, KpAtg14-Cerulean colocalized with YFP-KpAtg8, which was observed as foci (Fig. 5C). These results indicated that KpAtg14 functions at the preautophagosomal structure (PAS) and is necessary for bulk autophagy.

We further examined whether KpAtg14 is necessary for the recruitment of downstream ATG proteins for autophagosome formation. In *S. cerevisiae*, ScAtg5, which is required for lipidation of ScAtg8, functions downstream of ScAtg14 in the autophagosome formation, and deletion of the *ScATG14* gene causes a drastic decrease in the localization of ScAtg5 at the PAS (Suzuki et al. 2007). Upon the induction of bulk autophagy, the puncta of YFP-KpAtg8 were detected in both the WT and *Kpatg14Δ* strains. However, KpAtg5-Cerulean dots were observed only in the WT strain and ~20% of these puncta colocalized with YFP-KpAtg8 (Fig. 5D and E). These results strongly suggested that KpAtg14 is necessary for recruiting the downstream ATG proteins, similar to ScAtg14 during bulk autophagy.

Atg14 is an autophagy-specific subunit of the class III phosphatidylinositol 3-kinase (PI3-K) complex 1 (hereafter referred to as PI3KC1) and is an important determinant of the PI3KC1 function required for autophagy (Obara and Ohsumi 2011). *In S. cerevisiae*, the PI3KC1 is composed of ScAtg14, ScAtg6, ScVps34, and ScVps15, and ScAtg14 and ScAtg6 are conjugated to each other. To investigate whether KpAtg14 interacts with KpAtg6, we constructed the strain expressing KpAtg14-FLAG and KpAtg6-HA and performed a coimmunoprecipitation analysis. We found that KpAtg6-HA was precipitated by anti-FLAG antibodies (Fig. S3), suggesting that KpAtg14 forms a complex with KpAtg6.

### The C-terminal region of KpAtg14 is dispensable for autophagy

During the alignment analysis, we found that KpAtg14 and OpAtg14 had a longer C-terminal region with 100–200 amino acids than ScAtg14 and SpAtg14 (Fig. S1). The structural analysis by AlphaFold2 indeed indicated that KpAtg14 and OpAtg14 had a longer intrinsically disordered region (Fig. S4A–D), indicating that Atg14 of methylotrophic yeasts has a conserved unique function. Therefore, we constructed the *Kpatg14Δ* strain expressing a mutant KpAtg14 that lacked the C-terminal 84 amino acids (hereafter referred to as the *Kpatg14-ΔC84* strain) (Fig. S4E). We monitored bulk degradation in the *Kpatg14-ΔC84* strain expressing KpPgk1-CFP and found that the cleaved form of CFP was detected in a manner similar to the WT strain (Fig. S4F), indicating that the C-terminal region of KpAtg14 is not required for bulk-autophagy. We also investigated the pexophagic activity in the *Kpatg14-ΔC84* strain expressing KpPex11-pHluorin under macro- and micropexophagy-induced condition. Similar to the WT strain, the cleaved form of pHluorin was detected by immunoblot analysis under both macro- and micropexophagy-induced conditions (Fig. S4G and H), indicating that the C-terminal region of KpAtg14 is dispensable for autophagy.

## Discussion

In this study, we established a novel FACS-based screening system to identify genes required for pexophagy using the methylotrophic yeast *K. phaffii*. This method allowed us to sort ~50 000 transformants at one time, which has a much higher throughput than conventional plate-based assays that screen hundreds of transformants (Stasyk et al. 2008). In addition, this novel screening method also allows the identification of *ATGs* required for pexophagy under macropexophagy-induced conditions. To date, micropexophagy-induced conditions have been primarily used to identify the *ATGs* required for pexophagy as plate colony screening under macropexophagy-induced conditions has often yielded false positive results. We believe that the high-throughput FACS-based screening protocol for macropexophagy mutants established in this study will help in the identification of other novel genes necessary for pexophagy with a higher probability.

The newly identified *KpATG14* gene was found to be required not only for macropexophagy but also for micropexophagy (Figs 2 and 3) similar to the other eight *ATGs* that were discovered and characterized in previous studies (Table 4). Our immediate goal is to discover genes required solely for either macro- or micropexophagy. As of today, ~20 *ATGs* have been identified to be required for pexophagy and all of them are necessary for both macro- or micropexophagy pathways (Oku and Sakai 2016). As such, further work is needed to identify genes specific for one of the two pexophagy pathways. Identification of genes required for either macro- or micropexophagy enables us to get closer to understanding the physiological significance of the use of these two distinct pathways for peroxisome degradation by yeasts. To achieve this, improving the current FACS-based screening system might be an option. When combined with other techniques including gene expression microarrays (Gallardo and Behra 2013), FACS screenings can prove to be more powerful in identifying specific genes of interest.

KpAtg14 was newly added to a list of genes required for pexophagy and bulk autophagy in methylotrophic yeasts. Atg14 is a key factor in determining the function of the PI3KC1, which is one of the core components of the autophagosome component (Obara and Ohsumi 2011). The role of Atg14 on autophagy and its molecular characteristics have been investigated in the budding yeast *S. cerevisiae* (Obara et al. 2006) and the fission yeast *S. pombe* (Sun et al. 2013), as well as in other organisms (Itakura et al. 2008, Liu et al. 2017, 2020, Thompson et al. 2021). However, a homologous protein of Atg14 has not been discovered in *K. phaffii* for so long, despite a previous attempt (Farré et al. 2010). We believe that the reason for this is the longer C-terminal region that is present only in methylotrophic yeasts compared to other yeast species (Figs S1 and S4A–D). In mammalian Atg14, the Barkor/Atg14(L) autophagosome-targeting sequence (BATS) domain is conserved at the C-terminus and the domain plays a role in autophagosome targeting and membrane curvature sensing (Fan et al. 2011). However, the BATS domain was not found to be conserved in any methylotrophic yeasts (data not shown). This could be an indication that the C-terminus of Atg14 in the methylotrophic yeasts holds a specific role. Our previous studies have shown that the C-terminal region of CbHap3, a component of the multimeric transcription factor, the CbHap complex, is required for methanol-regulated gene expression, and that such a unique sequence was conserved among methylotrophic yeasts, but not in *S. cerevisiae* (Oda et al. 2015, 2016). However, deletion of the C-terminal region of KpAtg14 did not cause any effect on macropexophagy, micropexophagy or bulk autophagy in our experiments. Further analysis may be necessary to identify the specific functions of the C-terminal regions of KpAtg14 and OpAtg14.

OpAtg14 was identified by PSI-BLAST with KpAtg14, but not with ScAtg14 (Fig. S1), which prompted us to further investigate whether a homologous protein of Atg14 could be found in other organisms. A homology search by PSI-BLAST with KpAtg14 identified hypothetical proteins from seven organisms, namely *Candida arabinofermentans, Candida boidinii, Ogataea philodendri, Pachysolen tannophilus, Pichia kudriavzevii, Pichia membranifaciens* and *Wickerhamomyces pijperi* (synonym *Pichia pijperi*). Among the seven hypothetical proteins, those from *C. arabinofermentans, P. tannophilus* and *W. pijperi* could also be found by PSI-BLAST with Sctg14, but the others could not be, suggesting that amino acid sequences of unconventional yeasts like *K. phaffii* can have their homologous proteins in various yeasts, and further investigation could lead to a comprehensive understanding of specific genes.

In summary, our novel FACS-based screening method discovered the previously unidentified *KpATG14* gene in the methylotrophic yeast *K. phaffii*. Microscopic and immunoblot analyses revealed that KpAtg14 is required for both macro- and micropexophagy. Our results indicated that KpAtg14 plays a critical role in the complete enclosure of peroxisomes in the vacuole under micropexophagy-induced conditions. Furthermore, KpAtg14, like ScAtg14, recruits downstream ATG proteins and thus is required for bulk autophagic degradation. These results indicated that KpAtg14 has a conserved role in autophagic degradation among other eukaryotes.

## Supplementary Material

foae022_Supplemental_Files
