# Supplementary material for: A novel fluorescence-activated cell sorting (FACS)-based screening identified ATG14, the gene required for pexophagy in the methylotrophic yeast
Source: FEMS Yeast Res. 2024 Jul 18;24:foae022. doi: 10.1093/femsyr/foae022 (PMC11305268; doi:10.1093/femsyr/foae022)
Supplement: foae022_Supplemental_Files [file foae022_supplemental_files.zip › FEMS-Yeast-Res_FACS-Atg14_SupFigLegends_20240613_FINAL.docx]

**Supplementary figure legends**

**Fig. S1.** Comparison of amino acid sequences of Atg14 in *K. phaffi*, *O. polymorpha*, *S. cerevisiae* and *S. pombe*. Identical residues are indicated with filled boxes. Black arrows and underlined sequences indicate the cysteine repeat and predicted coiled-coil domains, respectively.

**Fig. S2.** Comparative visualization of coiled-coil predictions and the detection of stable single *α*-helices (SAH domains). Amino acid sequences of Atg14 in (a) *K. phaffi*, (b) *O. polymorpha*, (c) *S. cerevisiae* and (d) *S. pombe* were examined by Waggawagga. The plot combines both coiled-coil and SAH predictions in one graph and visualizes the probabilities over the length of the sequence. Filled areas in red and blue indicate the probability of the coiled-coil and SAH, respectively. Dashed lines in red and blue are thresholds of the coiled-coil and SAH, respectively.

**Fig. S3.** Immunoblot analyses of lysates of *K. phaffii* cells expressing KpAtg14-FLAG and KpAtg6-3xHA under the control of endogenous promoters, after immunoprecipitation. Protein samples were purified with anti-FLAG beads and subsequently detected with anti-FLAG and anti-HA antibodies.

**Fig. S4.** Deletion analysis of the C-terminal region of KpAtg14. (a)-(d) Predicted structure of Atg14 based on its amino acid sequence by AlphaFold2 (a. *K. phaffii*, b. *O. polymorpha*, c. *S. cerevisiae*, d. *S. pombe*). Cysteine repeat domain, coiled-coil domain and IDR are highlighted in red, green and blue, respectively. (e) Immunoblot analysis of the wild-type strains expressing KpAtg14ΔC84-FLAG and KpAtg14-FLAG during bulk autophagy-induced conditions. (f) Immunoblot analysis of FLAG-tagged KpAtg14 and KpAtg14ΔC84 in *K. phaffii* wild-type cells under bulk autophagy-induced conditions. (g) Immunoblot analysis of FLAG-tagged KpAtg14 and KpAtg14ΔC84 in *K. phaffii* wild-type cells under macropexophagy-induced conditions. (h) Immunoblot analysis of FLAG-tagged KpAtg14 and KpAtg14ΔC84 in *K. phaffii* wild-type cells under micropexophagy-induced conditions. (f)-(h) ΔC84, FL and Vec indicate KpAtg14ΔC84, KpAtg14-FLAG and empty vector, respectively.
