# Supplementary figures and images for: A novel fluorescence-activated cell sorting (FACS)-based screening identified ATG14, the gene required for pexophagy in the methylotrophic yeast
Source: FEMS Yeast Res. 2024 Jul 18;24:foae022. doi: 10.1093/femsyr/foae022 (PMC11305268; doi:10.1093/femsyr/foae022)

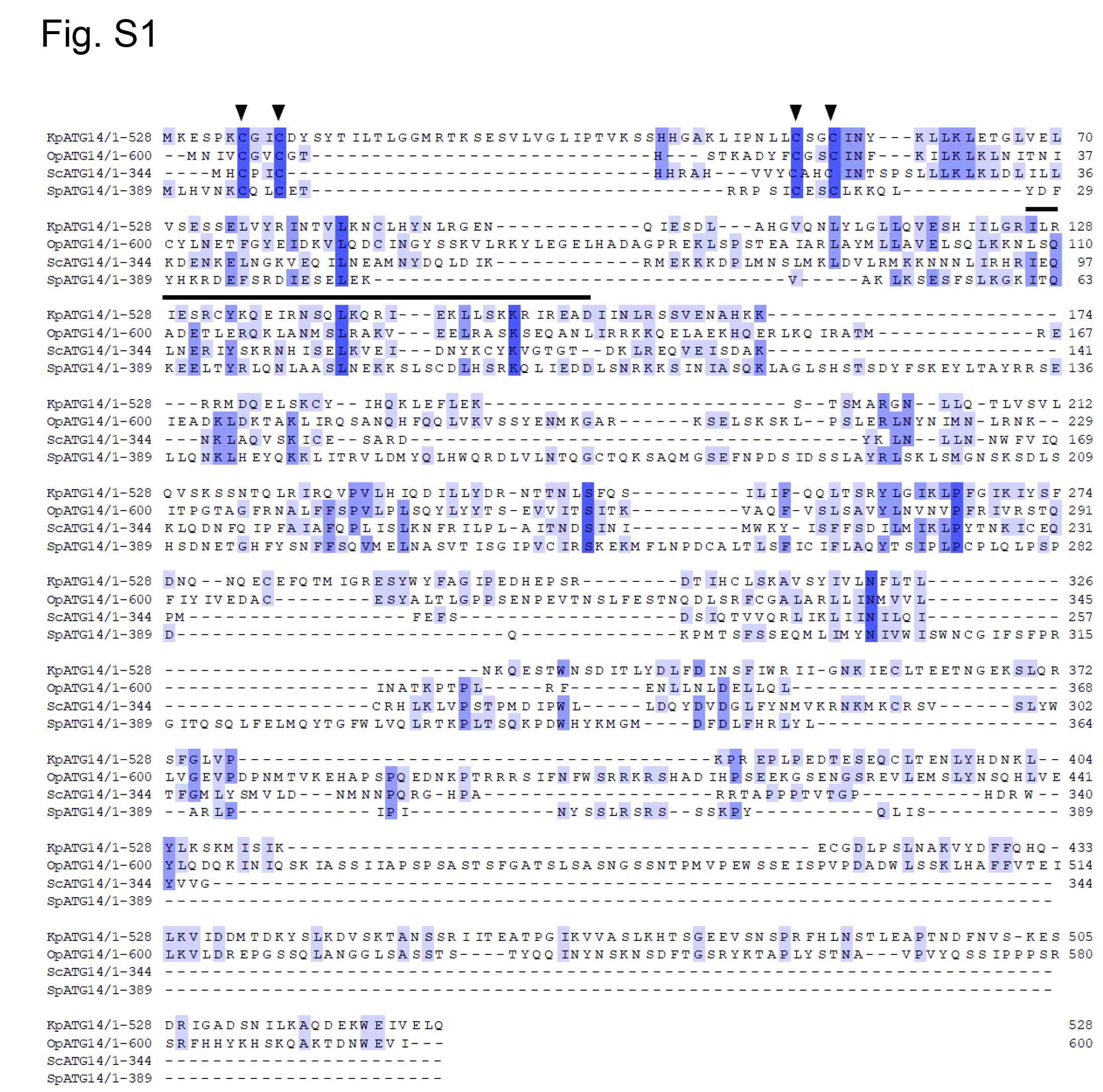

Supplement: foae022_Supplemental_Files [file foae022_supplemental_files.zip › Figure_S1.tif]

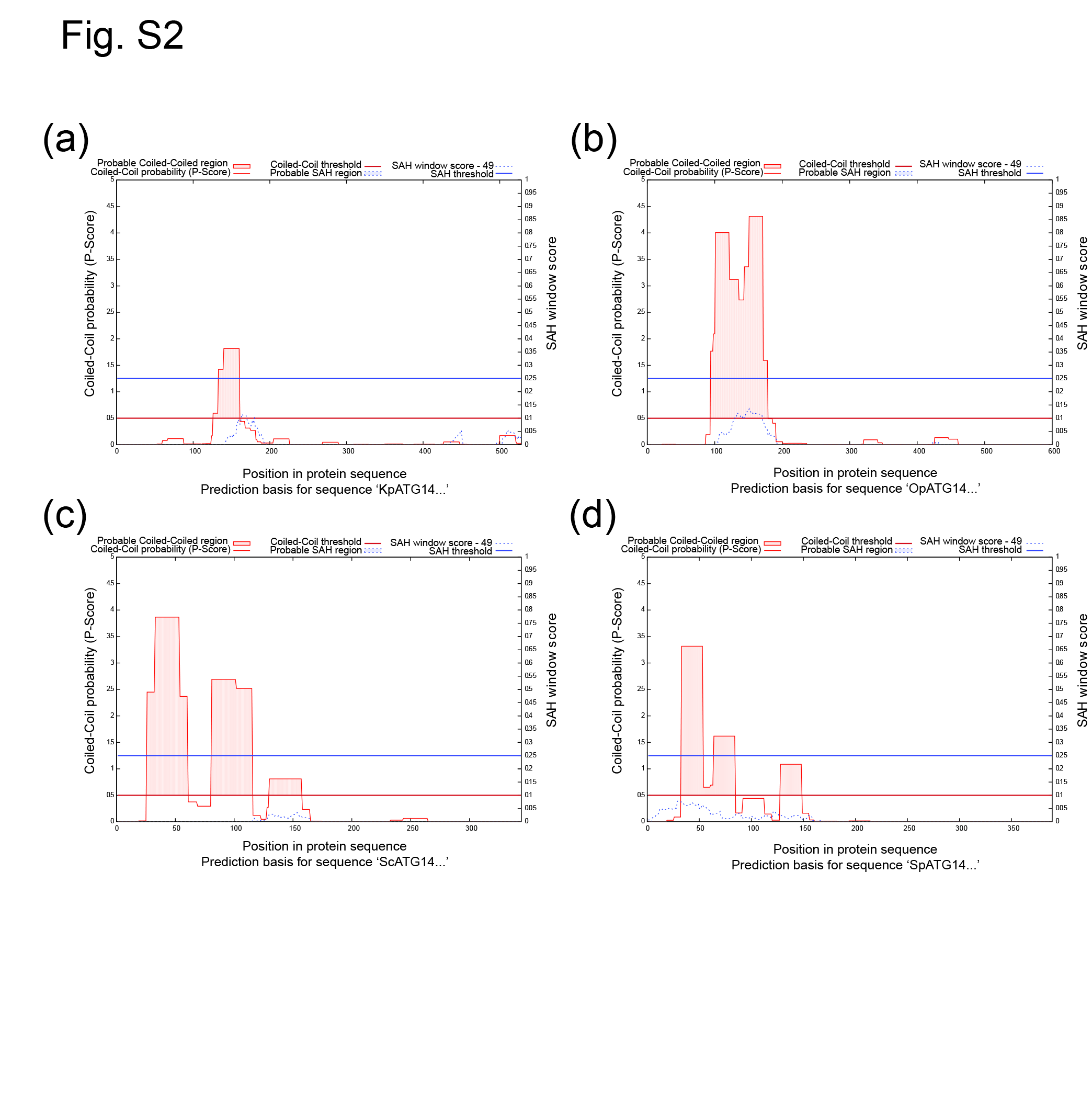

Supplement: foae022_Supplemental_Files [file foae022_supplemental_files.zip › Figure_S2.tif]

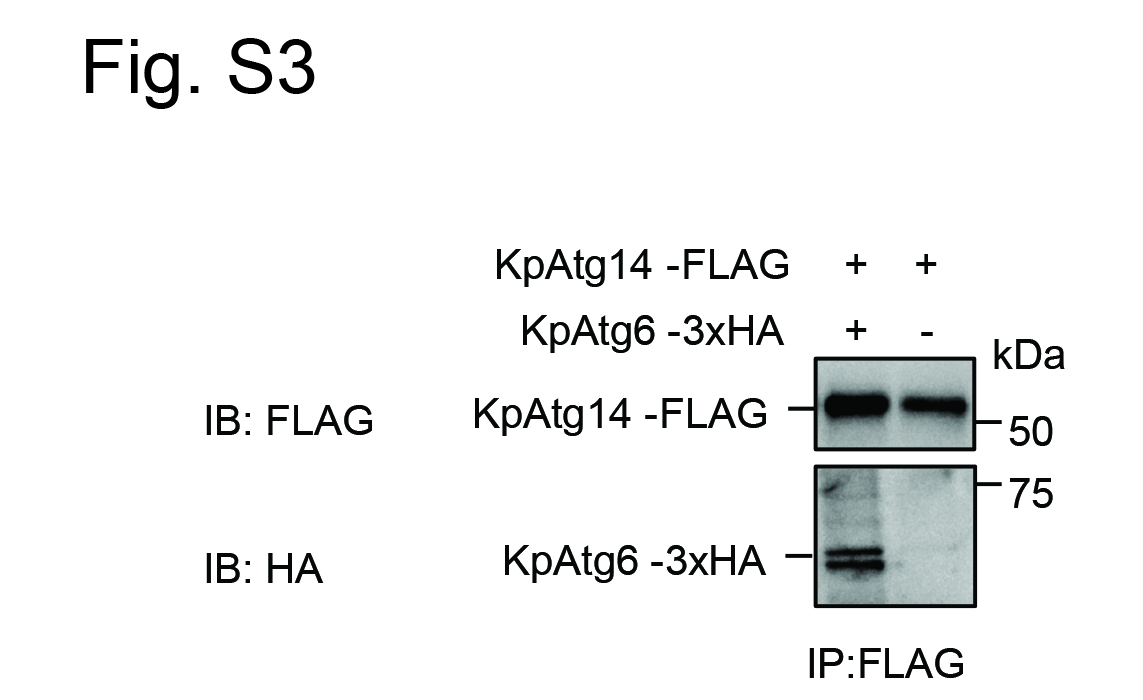

Supplement: foae022_Supplemental_Files [file foae022_supplemental_files.zip › Figure_S3.tif]

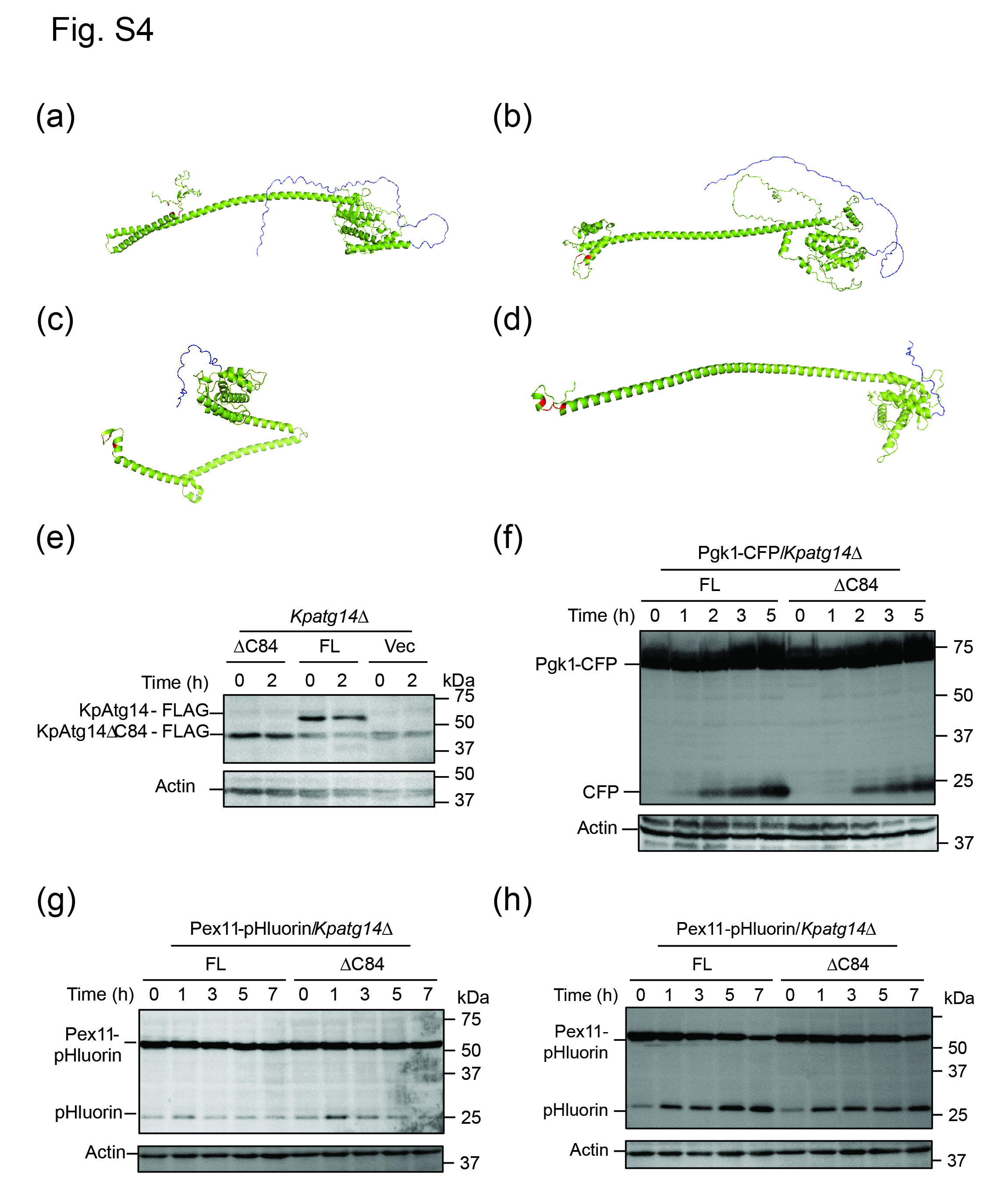

Supplement: foae022_Supplemental_Files [file foae022_supplemental_files.zip › Figure_S4.tif]
